# *Glossogyne tenuifolia* Extract Inhibits TNF-α-Induced Expression of Adhesion Molecules in Human Umbilical Vein Endothelial Cells via Blocking the NF-κB Signaling Pathway

**DOI:** 10.3390/molecules200916908

**Published:** 2015-09-17

**Authors:** Chin-Feng Hsuan, Hsia-Fen Hsu, Wei-Kung Tseng, Thung-Lip Lee, Yu-Feng Wei, Kwan-Lih Hsu, Chau-Chung Wu, Jer-Yiing Houng

**Affiliations:** 1 Institute of Biotechnology and Chemical Engineering, I-Shou University, Kaohsiung 84001, Taiwan; E-Mail: calvin.hsuan@msa.hinet.net; 2 Division of Cardiology, Department of Internal Medicine, E-Da Hospital/I-Shou University, Kaohsiung 82445, Taiwan; E-Mails: arthurtseng@seed.net.tw (W.-K.T.); lip1969@hotmail.com (T.-L.L.); ed103914@edah.org.tw (K.-L.H.); 3 Department of Nutrition, I-Shou University, Kaohsiung 82445, Taiwan; E-Mail: fen153848@yahoo.com.tw; 4 Division of Pulmonary Medicine, Department of Internal Medicine, E-Da Hospital/I-Shou University, Kaohsiung 82445, Taiwan; E-Mail: yufeng528@gmail.com; 5 Division of Cardiology, Department of Internal Medicine, National Taiwan University Hospital, Taipei 10002, Taiwan; E-Mail: chauchungwu@ntu.edu.tw

**Keywords:** *Glossogyne tenuifolia*, luteolin, luteolin-7-glucoside, adhesion molecule, nuclear factor-κB

## Abstract

Chronic inflammation plays a pivotal role in the development of atherosclerosis, where the pro-inflammatory cytokine-induced expression of endothelial adhesion molecules and the recruitment of monocytes are the crucial events leading to its pathogenesis. *Glossogyne tenuifolia* ethanol extract (GTE) is shown to have potent anti-inflammatory and antioxidant activities. We evaluated the effects of GTE and its major components, luteolin (lut), luteolin-7-glucoside (lut-7-g), and oleanolic acid (OA) on TNF-α-induced expression of adhesion molecules in human umbilical vein endothelial cells (HUVECs). The results demonstrated that GTE, lut, and lut-7-g attenuated the expression of intercellular adhesion molecule-1 (ICAM-1) and vascular cell adhesion molecule-1 (VCAM-1) in TNF-α-activated HUVECs, and inhibited the adhesion of monocytes to TNF-α-activated HUVECs. The TNF-α-induced mRNA expression of ICAM-1 and VCAM-1 was also suppressed, revealing their inhibitory effects at the transcriptional level. Furthermore, GTE, lut, and lut-7-g blocked the TNF-α-induced degradation of nuclear factor-κB inhibitor (IκB), an indicator of the activation of nuclear factor-κB (NF-κB). In summary, GTE and its bioactive components were effective in preventing the adhesion of monocytes to cytokine-activated endothelium by the inhibition of expression of adhesion molecules, which in turn is mediated through blocking the activation and nuclear translocation of NF-κB. The current results reveal the therapeutic potential of GTE in atherosclerosis.

## 1. Introduction

Atherosclerosis is a chronic inflammation process involving interactions between modified lipoproteins, cytokines, monocyte-derived macrophages, T-cells, and the cellular components of arterial walls [1,2,3]. The recruitment of monocytes to the arterial wall constitutes the early essential event of atherosclerosis. This process is mediated by the interaction between the adhesion molecules expressed on the endothelium and its specific ligands on monocytes. Vascular endothelium, upon stimulation by the oxidized low-density lipoprotein (LDL) and pro-inflammatory cytokines, up-regulate the expression of adhesion molecules, including vascular cell adhesion molecule-1 (VCAM-1) and intercellular adhesion molecule-1 (ICAM-1) [4]. Subsequently, it promotes the adherence of circulating monocytes to the vascular endothelium and facilitates their migration to the subendothelial space [5,6].

Nuclear factor-κB (NF-κB), a transcription factor, appears to be the major intracellular signaling peptide mediating the increased expression of endothelial adhesion molecules, induced by oxidized LDL and cytokines [7,8,9]. NF-κB is bound to the nuclear factor-κB inhibitor (IκB) and is retained in the cell cytoplasm, while not activated. Once the cell is stimulated, IκB is phosphorylated by specific kinases and degraded rapidly, subsequently releasing the NF-κB. The released NF-κB translocates to the nucleus, where it regulates the gene expression [10]. Monocyte-derived pro-inflammatory cytokines, such as, tumor necrosis factor-α (TNF-α) and interleukin-1 (IL-1), have been demonstrated to increase the expression of ICAM-1 and VCAM-1 via the canonical NF-κB signaling pathway [11].

Chemicals modulating the expression of adhesion molecules have the potential to prevent the progression of atherosclerosis. Recently, greater emphasis has been placed on the anti-inflammatory and anti-atherogenic effects of plant derived dietary supplements and phytochemicals. Epidemiological and clinical research revealed that increased intake of fruits, vegetables, and drinks rich in polyphenols, like resveratrol, proanthocyanidins, and flavonoids, decreased the risk of cardiovascular disease [12,13,14,15,16,17]. The protective effects are believed to be related to the inhibition of the expression of adhesion molecules. *In vivo* studies have shown that polyphenols, such as, resveratrol form red wine, catechins from green tea, and other plant flavonoids, including luteolin and apigenin, were proved to inhibit the expression of adhesion molecules and the adhesion of monocytes to the endothelium [18,19,20,21].

*Glossogyne tenuifolia* (GT), a perennial plant, distributed in Southern Asia and Australia, is used to make a traditional antipyretic, hepatoprotective, and anti-inflammatory herbal tea in Penghu, Taiwan [22]. Three major bioactive ingredients of GT have been reported [23], of which luteolin (lut) and luteolin-7-glucoside (lut-7-g) are flavonoids, while oleanolic acid (OA) is a triterpenoid. Previous studies on GT ethanol extract (GTE) and its major flavonoids, lut-7-g and lut, demonstrated the antioxidant [23,24,25], antiviral [26], anti-osteoclastogenic [27] and immunomodulatory properties [28] in addition to its cytotoxicity on several human cancer cell lines [23]. It is also known to prevent the oxidation of LDL [24], and protect against the endothelial injury by inhibiting the free reactive oxygen species (ROS) formation [29]. *In vitro* studies have revealed that GTE, lut, lut-7-g, and OA, inhibited the inflammatory mediator production [26,28,30]. Wu *et al.* found that GTE and its active components attenuated the inflammatory mediator synthesis through the NF-κB pathway [30].

Owing to its constituent polyphenolic flavonoids, GTE exhibit good anti-inflammatory and antioxidant activity, thus providing a potential therapeutic remedy to attenuate the vascular inflammation and prevent atherosclerosis. However, until now, this potential remains unexplored. The present study was designed to examine the effects of GTE and its bioactive components, lut, lut-7-g, and OA on cytokine-induced expression of adhesion molecules in human umbilical vein endothelial cells (HUVECs) and to delineate the underlying mechanisms.

## 2. Results and Discussion

### 2.1. Cell Viability

**Figure 1:**
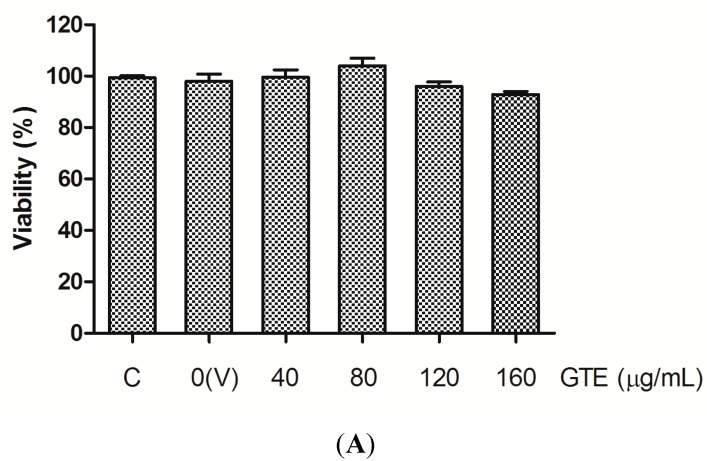

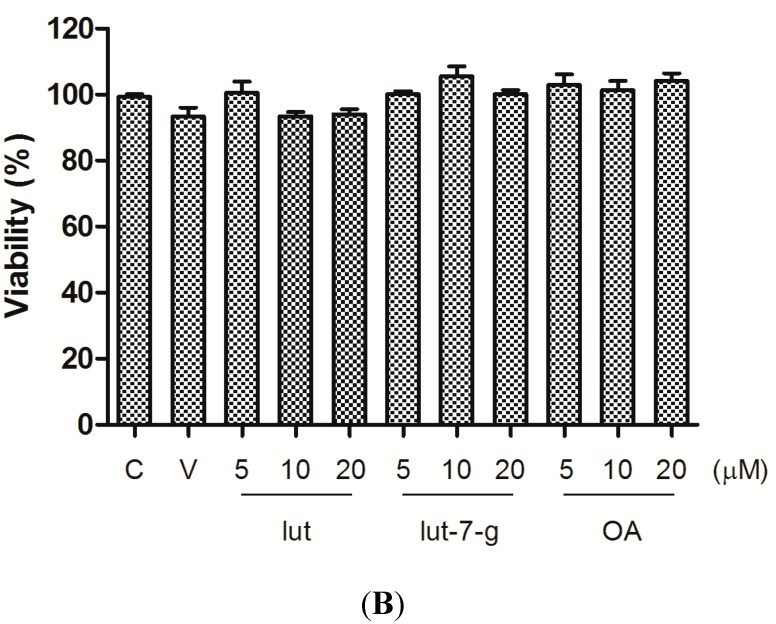
Effect of GTE, lut, lut-7-g, and OA on cell viability. MTT assay was performed to assess the cytotoxicity of GTE, lut, lut-7-g, and OA on HUVECs. HUVECs were treated with medium only (Control, denotes as “C”), vehicle only (DMSO, 0.25% for GTE group and 0.1% for pure compounds group; denotes as “V”), GTE (40, 80, 120, 160 μg/mL), lut (5, 10, 20 μM), lut-7-g (5, 10, 20 μM), and OA (5, 10, 20 μM) for 24 h. The cell viability was not affected after incubation for 24 h with, (**A**) GTE at concentrations up to 160 μg/mL; and (**B**) lut, lut-7-g, and OA at concentrations up to 20 μM.

At first, the cytotoxicity of GTE, lut, lut-7-g, and OA on HUVECs were assessed using the MTT (3-(4,5-dimethylthiazol-2-yl)-2,5-diphenyltetrazolium bromide) assay. The cell viability at 24 h did not decrease after incubation with GTE at concentrations up to 160 μg/mL and with lut, lut-7-g, or OA, at concentrations up to 20 μM (Figure 1), indicating that GTE, lut, lut-7-g, and OA were not cytotoxic to HUVECs within the concentration ranges tested.

### 2.2. Effects of GTE, Lut, Lut-7-g, and OA on Adhesion of THP-1 Monocytes to HUVECs

**Figure 2:**
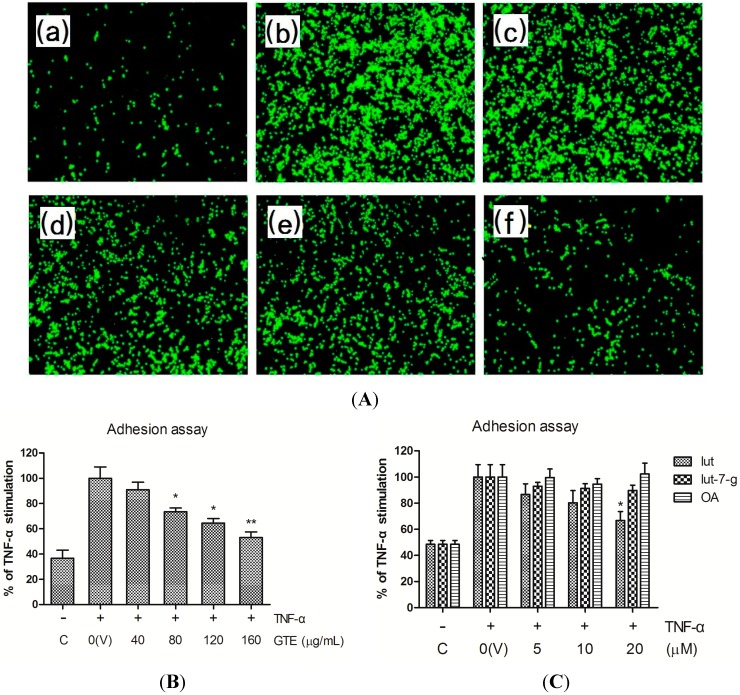
Inhibitory effects of GTE, lut, lut-7-g, and OA on adhesion of THP-1 monocytes to TNF-α-activated HUVECs. HUVECs were pretreated with vehicle only (V), GTE (40, 80, 120 and 160 μg/mL), lut (5, 10, 20 μM), lut-7-g (5, 10, 20 μM), and OA (5, 10, 20 μM) for 2 h, and were then activated by TNF-α for 6 h. After treatment, confluent HUVECs were co-cultured with calcein-AM-labeled THP-1 monocytes for 30 min. Cell images were collected by a fluorescence microscope and quantified using a fluorescence microplate reader. (**A**) Representative images showing the inhibition of THP-1 cells to TNF-α-activated HUVECs after pretreatment with the indicated doses of GTE. (**a**) HUVECs treated with medium only (control, C), (**b**–**f**) TNF-α-activated HUVECs, pretreated with 0 (vehicle only, V), 40, 80, 120, and 160 μg/mL of GTE for 2 h; (**B**) Pretreatment of HUVECs with GTE; (**C**) Pretreatment of HUVECs with lut, lut-7-g, and OA. A significant difference from the vehicle was indicated as * *p* < 0.05, or ** *p* < 0.01 (Student’s *t*-test).

The monocyte adhesion assay was performed to explore the effects of GTE and its bioactive compounds on monocyte-endothelial cell interactions. At baseline, human acute monocytic leukemia THP-1 cell line showed minimal adherence to the unstimulated HUVECs. After treatment of HUVECs with TNF-α (10 ng/mL) for 1 h, the adhesion of THP-1 to HUVECs increased markedly. Adhesion of THP-1 to TNF-α-stimulated HUVECs was inhibited by pretreating the HUVECs with GTE in a dose-dependent manner (Figure 2A,B). Pretreatment with lut significantly inhibited the adhesion of THP-1 to TNF-α-stimulated HUVECs at a dose of 20 μM. Lut-7-g exhibited a trend of inhibition of adhesion, although not significant. However, OA did not inhibit the adhesion of THP-1 to TNF-α activated HUVECs (Figure 2C).

### 2.3. Effects of GTE, Lut, Lut-7-g, and OA on TNF-α-Induced Adhesion Molecule Expression

**Figure 3:**
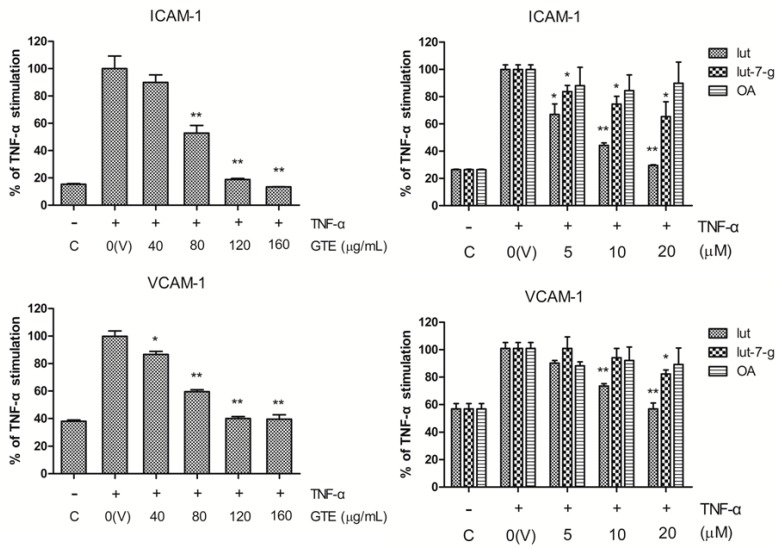
Inhibitory effects of GTE, lut, lut-7-g, and OA on the expression of ICAM-1 and VCAM-1 in TNF-α-activated HUVECs. TNF-α-activated HUVECs were pretreated with vehicle only (V), GTE (40, 80, 120, 160 μg/mL), lut (5, 10, 20 μM), lut-7-g (5, 10, 20 μM), and OA (5, 10, 20 μM) for 2 h. The expression of ICAM-1 and VCAM-1 on plasma membranes of HUVECs was measured by ELISA. A significant difference from the vehicle was indicated as, * *p* < 0.05, or ** *p* < 0.01 (Student’s *t*-test).

Previous studies have revealed that adhesion molecules, such as ICAM-1 and VCAM-1, are involved in the adhesion of monocytes to cytokine-activated endothelial cells [1,2,3,4]. Treatment of HUVECs with TNF-α for 6 h markedly upregulated the cell surface expression of ICAM-1 and VCAM-1. Pretreatment of the cells with GTE, lut, lut-7-g, and OA were performed to examine their inhibitory effects on TNF-α-induced expression of ICAM-1 and VCAM-1. As shown in Figure 3, pretreatment of HUVECs with GTE significantly inhibited the cell surface expression of both adhesion molecules in a dose-dependent manner. Lut and lut-7-g induced significant dose-dependent inhibition of ICAM-1 expression, especially at doses higher than 5 μM. Furthermore, the expression of VCAM-1 was markedly inhibited by lut and lut-7-g (above 10 μM). In contrast, the triterpenoid OA failed to inhibit the expression of ICAM-1 and VCAM-1.

### 2.4. Effects of GTE, Lut, and Lut-7-g on the mRNA Level of Adhesion Molecules

**Figure 4:**
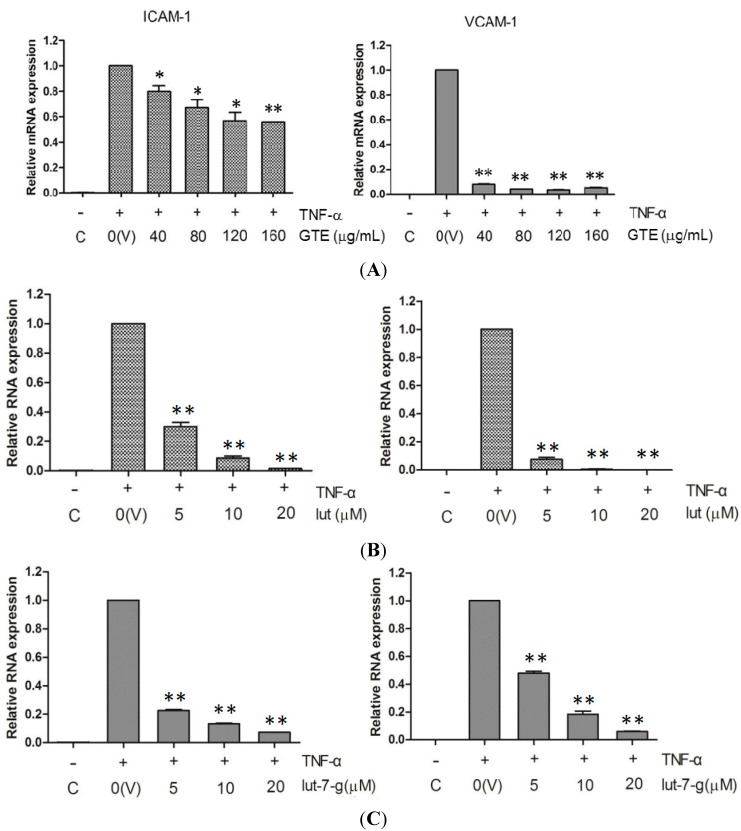
Inhibition of TNF-α-induced mRNA expression of adhesion molecules by: (**A**) GTE; (**B**) lut; and (**C**) lut-7-g. TNF-α-activated HUVECs were pretreated with vehicle only (V), GTE (40, 80, 120, 160 μg/mL), lut (5, 10, 20 μM), lut-7-g (5, 10, 20 μM), and OA (5, 10, 20 μM) for 2 h. Real-time RT-PCR analysis was performed to analyze the expression level of mRNA of ICAM-1 and VCAM-1. A significant difference from the vehicle was indicated as, * *p* < 0.05, or ** *p* < 0.01 (Student’s *t*-test).

The inhibitory effects of GTE, lut, and lut-7-g on TNF-α-induced adhesion molecules were further examined in terms of their mRNA expression. As shown in Figure 4, the signals for mRNA expression of ICAM-1 and VCAM-1 were weak for the un-stimulated cells, but it increased greatly in TNF-α-stimulated (10 ng/mL for 6 h) HUVECs. Pretreatment of HUVECs with GTE, lut, and lut-7-g markedly suppressed the TNF-α-induced mRNA expression of ICAM-1 and VCAM-1. These data indicate that GTE, lut, and lut-7-g inhibit the cell surface expression of ICAM-1 and VCAM-1 via suppression at HUVECs’ gene transcription level.

### 2.5. Effects of GTE, Lut, and Lut-7-g on the Degradation of IκBα

**Figure 5:**
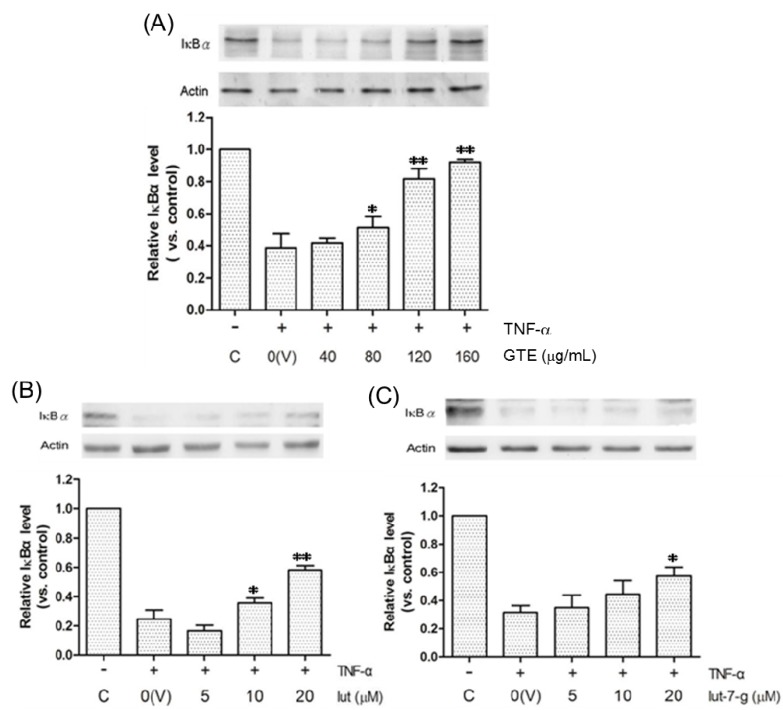
Inhibitory effects of (**A**) GTE; (**B**) lut; and (**C**) lut-7-g on the degradation of IκBα. HUVECs were pretreated with vehicle only (V), GTE (40, 80, 120, 160 μg/mL), lut (5, 10, 20 μM), lut-7-g (5, 10, 20 μM) and OA (5, 10, 20 μM) for 2 h, and were then activated by TNF-α for 1 h. The whole-cell lysates were subjected to Western blot assays, using β-actin as an internal control. The relative density of the proteins was determined by densitometric analysis. A significant difference from the vehicle was indicated as, * *p* < 0.05, or ** *p* < 0.01 (Student’s *t*-test).

In this study, IκBα (α isoform of IκB) was used as an indicator of the status of NF-κB activation. Generally, NF-κB is bound to IκB and is retained in the cellular cytoplasm in an inactivated state. Once the cell is stimulated by TNF-α, IκB is phosphorylated and degraded rapidly. NF-κB is then released and is translocated into the nucleus, where it regulates the gene expression [10]. Therefore, decreased IκB level indicates increased NF-κB activity. Figure 5 shows that the treatment of HUVECs with TNF-α for 1 h increased the degradation of IκBα. GTE blocked TNF-α-induced degradation of IκBα, implying that GTE inhibited TNF-α-induced activation and nuclear translocation of NF-κB. Additionally, lut, and lut-7-g also exhibited this suppression effect (Figure 5).

Several investigations demonstrated GTE could inhibit the production of inflammatory mediators [26,28,30]. However, not all inflammatory mediators were consistently inhibited by its active compounds, lut, lut-7-g, and OA, in different cell types. In the present study, we further demonstrated the anti-atherogenic effect of GTE, lut, and lut-7-g by investigating their inhibitory effects on the expression of adhesion molecules in HUVECs, which was mediated via blocking the NF-κB signaling pathway. These results suggest that the flavonoids (lut and lut-7-g), rather than triterpenoid (OA) mediate the potent anti-atherogenic effects of GT. As reported, GTE possessed significant antioxidant activity [24,25], which was correlated with the polyphenol and flavonoid contents in the various partitions of GT [23,31]. The observation of the present study is consistent with the literature, where the antioxidant activity of many edible plant extracts increases with its flavonoid content [32]. Hsu *et al.* identified the three main ingredients, OA, lut, and lut-7-g, from the fraction which possessed the highest antioxidant activity [23]. Among them, the antioxidant activity of OA was low. In contrast, lut and lut-7 exhibited good antioxidant activity, suggesting that the antioxidant activity of GT is mainly attributed by the flavonoid components.

Lut is one of the flavonoids contained in vegetables and many medicinal plants [33]. Some epidemiological studies indicate that a high dietary luteolin intake was associated with a lower risk of cardiovascular disease [34,35], which is probably linked to its high antioxidant and anti-inflammatory activities [33,36] which, in turn, prevent the development of atherosclerosis. Lut is present in plants as glycone and as glycosides. Lut glycosides are cleaved to their aglycones in the intestinal mucosa [36]. Lut and its glycosides have a dihydroxy structure in 3′ and 4′ positions of the B ring, which serves as a hydrogen (electron) donor to aid in the scavenging of ROS [37,38]. Another structural feature responsible for the antioxidant activity is the presence of a 2,3-double bond in conjugation with a 4-oxo group in the C-ring, which serves to chelate transition metals that may induce oxidative damage [39].

Lut suppresses the expression of pro-inflammatory cytokines and chemokines via blocking the transcription factors such as NF-κB and activating protein-1 (AP-1) [33,40]. Lut also inhibits pro-inflammatory enzymes, including cyclooxygenases, lipoxygenases and inducible nitric oxide synthase [41,42]. Additionally, lut was demonstrated to attenuate the pro-inflammatory cytokines induced expression of adhesion molecules in endothelial cells [20]. One important mechanism for the down-regulation of adhesion molecules was the modulation of the NF-κB signaling pathway by lut [21,43].

Inflammatory cytokines, such as TNF-α and IL-1β, may induce cellular synthesis of ROS [44,45]. ROS play a vital role as signaling molecules in the cytokine triggered NF-κB activation [46,47]. Based on the evidences observed in this study, we suppose that the anti-atherogenic effect of GTE and its active components, lut, and lut-7-g, may be attributed to their antioxidant activity.

## 3. Experimental Section

### 3.1. Materials and Reagents

The raw materials of *G. tenuifolia* (GT) were bought from an herb store in Penghu Island, Taiwan, the integrity of which was assessed by DNA sequence identification [23]. Penicillin, streptomycin, dimethyl sulfoxide (DMSO), and MTT were purchased from Sigma Chemicals Co. (St Louis, MO, USA). Human recombinant TNF-α was obtained from Peprotech (Rocky Hill, NJ, USA). All other chemicals used were of reagent or analytical grade.

### 3.2. Preparation of GTE Extract and Separation of Its Main Ingredients

The preparation of GTE, lut, lut-7-g, and OA were performed, as described previously [23]. In brief, the dry whole plant materials of GT (5.3 kg) were extracted three times, with 20 L of ethanol for one day each time. After filtration by medicinal gauze, the filtrates were concentrated with a vacuum evaporator and dried with a freeze-drier. The weight of this GTE was 777 g, and the yield was 14.7%. The chemical compositions of GTE were analyzed by HPLC (Hitachi, Tokyo, Japan), UPLC-ESI-MS (Thermo Scientific, Germering, Germany) (Figure S1) and gas chromatography-mass spectrometry (Perkin Elmer, Waltham, MA, USA) (Figure S2 and Table S1).

The GTE (400 g) was further extracted by ethyl acetate (EA). The EA extract (61.5 g) was separated by a silica gel chromatography column (760 mm length, 120 mm diameter, packed with 2.5 kg silica gel (Si-60, 40–63 μm, Merck Co., Kenilworth, NJ, USA). The samples were eluted sequentially with 1.5 L gradient of *n*-hexane/EA (from 90:10, 80:20–0:100), followed by methanol up to 100%. After the operation, 15 fractions were collected. The OA was separated from Fraction 6 and its dry weight was 6.13 g. Lut was obtained from Fractions 8, 9, and 10, and its dry weight in these fractions was 2.31 g, 1.01 g and 1.13 g, respectively. The lut-7-g was separated from Fraction 12 and its dry weight was 0.31 g. These three compounds were identified by direct comparison of their optical rotation, IR spectra, ^1^H-, ^13^C-NMR and low-resolution EIMS with authentic samples. These compounds were dissolved in DMSO to make fresh stock solutions before each assay. The final culture concentration of DMSO was 0.25% for GTE and 0.1% for pure compound.

### 3.3. Cell Cultures

Primary HUVECs were obtained from The American Type Culture Collection (ATCC, Manassas, VA, USA) and maintained in endothelial cell growth medium 211K-500 (Cell Application, San Diego, CA, USA) at 37 °C under 5% CO_2_ atmosphere. Cells were seeded at a density of 2 × 10^4^ cells/well into a 96-well plate coated with gelatin (Sigma) and used for adhesion assay or immunoassay. Confluent HUVECs on 3.5 cm, gelatin-coated dishes were prepared for RNA or protein extraction. HUVECs with passage between four and six were used in the study.

Human acute monocytic leukemia THP-1 cell line was obtained from Bioresource Collection and Research Center (BCRC, Hsinchu, Taiwan) and cultured in RPMI-1640 (Sigma) supplemented with 10% fetal bovine serum (FBS) (Biological Industries, Beit HaEmek, Israel), 1.5 g/L sodium bicarbonate, 4.5 g/L glucose, 10 mM HEPES and 1.0 mM sodium pyruvate, 0.05 mM 2-mercaptoethanol, 100 U/mL penicillin, and 10 μg/mL streptomycin at 37 °C under 5% CO_2_ atmosphere. THP-1 cells of passage 10 to 15 were used for the adhesion assay.

### 3.4. MTT Assay for Cell Viability

HUVECs were seeded into 96-well plates at 2 × 10^4^ cells per well and treated with the indicated concentration of GTE and pure compounds. The cells were cultivated at 37 °C with 5% CO_2_ and 95% air, at 100% relative humidity. After 24 h incubation, the medium solution was removed. An aliquot of 10 μL, 5 mg/mL MTT was added to the plates and the cells were incubated for 4 h. After removing medium solution, 100 µL of DMSO were added to each well to dissolve the formazan crystals formed, which was shaken until the crystals dissolved. The cytotoxicity against HUVECs was determined by measuring the absorbance of the converted dye at a wavelength of 570 nm in an ELISA reader (Tecan Sunrise, Mannedorf, Switzerland). Cell viability (% of control) was calculated as (experimental OD/control OD) × 100%.

### 3.5. Monocyte Adhesion Assay

HUVECs were starved in a serum-free endothelial cell growth medium 211F-500 (Cell Application) for 1 h and treated with vehicle (DMSO) only, GTE (40, 80, 120, 160 μg/mL), lut (5, 10, 20 μM), lut-7-g (5, 10, 20 μM), and OA (5, 10, 20 μM) for 2 h. Cells were then stimulated with 10 ng/mL TNF-α for 6 h. After treatment, the medium was removed, and confluent HUVECs in 96-well plates were washed three times. 8 × 10^4^ THP-1 cells labeled with 5 μM calcein-AM (Invitrogen, Waltham, MA, USA) were seeded onto confluent HUVECs and co-cultured for 30 min at 37 °C under 5% CO_2_ atmosphere. Non-adherent THP-1 cells were removed by washing with phosphate buffered saline (PBS) three times. Cell images were collected by a fluorescence microscope (Carl Zeiss, Oberkochen Germany) and quantified using a fluorescence microplate reader (Bio-Tek Synergy HT, Winooski, VT, USA) at an excitation wavelength of 490 nm and an emission wavelength of 525 nm.

### 3.6. Cell Surface Immunoassay

Surface expression of cell adhesion molecules on HUVECs was measured by enzyme-linked immunosorbent assay (ELISA), as described previously [48]. Briefly, serum-starved HUVECs were pretreated with vehicle (DMSO) only, GTE (40, 80, 120, 160 μg/mL), lut (5, 10, 20 μM), lut-7-g (5, 10, 20 μM), and OA (5, 10, 20 μM) for 2 h and then stimulated with 10 ng/mL TNF-α for 6 h. After removal of the culture supernatants, the cells were fixed with 4% paraformaldehyde for 20 min at 4 °C and blocked with 0.5% bovine serum albumin (BSA, Sigma) for 1 h to prevent non-specific bindings. The cell monolayer was stained with mouse monoclonal antibodies against ICAM-1 (1:1000, Abcam, Cambridge, MA, USA) or VCAM-1 (1:1000, Abcam) at 4 °C overnight and then washed with PBS, followed by incubation with alkaline phosphatase conjugated goat anti-mouse antibodies (1:1000, Santa Cruz, TX, USA) for 1 h. After washing, *p*-nitrophenyl phosphate (PNPP, Invitrogen) was added and the absorbance was measured with an ELISA reader at a wavelength of 405 nm.

### 3.7. Real-Time RT-PCR Analysis

Total RNA was extracted from the treated HUVECs using Trizol reagent (Invitrogen). First-stand cDNA was synthesized from 5 μg total RNA of each sample using GoScript Reverse Transcription System (Promega, San Luis Obispo, CA, USA) according to the manufacturer’s instruction. The quantitative real-time PCR assay was performed using 7500 Real-Time PCR System (Applied Biosystems, Foster City, CA, USA) in triplicate in a 20 μL reaction volume containing 1.5 μL cDNA, 0.3 μM each primer, and 1× SYBR qPCR Master Mix (Fermentas). Primer sequences of human-ICAM-1 (forward: 5′-TCACGGAGCTCCCAGTCCTAA-3′, reverse: 5′-AAAGGCAGGTTGGCCAATGA-3′), human-VCAM-1(forward: 5′-CGAAAGGCCCAGTTGAAGGA-3′, reverse: 5′-GAGCACGAGAAGCTCAGGAGAAA-3′), and human-GAPDH (forward: 5′-GCACCGTCAAGGCTGAGAAC-3′, reverse: 5′-TGGTGAAGACGCCAGTGGA-3′) were used, as described previously [49]. The reaction conditions were 95 °C for 10 min, followed by 40 cycles of 95 °C for 15 s, and a final extension of 60 °C for 10 min. GAPDH was used as an endogenous control to normalize input amount of each sample. The relative expression levels of ICAM-1 and VCAM-1 were analyzed using the comparative Ct method.

### 3.8. Western Blot Analysis

Treated HUVECs were scraped off from the dish and lysed with RIPA buffer (Roche, Switzerland), which consists of 50 mM Tris-HCl (pH 7.4), 150 mM NaCl, 0.5% sodium deoxycholate, 1% NP-40, 1% sodium dodecyl sulfate (SDS), and protease inhibitor cocktail, at 4 °C overnight. The protein concentration of the cell lysate was quantified using the Bradford Protein Assay Kit (Bio-Rad, California, CA, USA). Equal amounts of protein from each sample were applied to a 10% SDS-polyacrylamide gel electrophoresis and transferred to a polyvinylidene fluoride (PVDF) membrane (Bio-Rad). The membrane was blocked with 5% skim milk and stained with rabbit polyclonal antibodies against IκBα (1:1000, Millipore, Billerica, MA, USA) or mouse monoclonal antibodies against β-actin (1:5000, Millipore) at 4 °C overnight. Further incubation for 1 h was performed using appropriate horseradish peroxidase conjugated secondary antibodies (Jackson Immunoresearch, West Grove, PA, USA), such as goat anti-rabbit antibodies (1:5000) for IκBα, and goat anti-mouse antibodies (1:5000) for β-actin. Bound antibodies were detected via Immobilon Western chemiluminescent HRP substrate (Millipore) and analyzed by densitometric analysis (UVP BioSpectrum 500 Imaging System, Upland, CA, USA).

### 3.9. Statistical Analysis

Data of each result were obtained from three to five independent experiments, each experiment in triplicate. Statistical differences were analyzed by Student’s *t*-test (* *p* < 0.05, ** *p* < 0.01). The experimental data were analyzed using Microsoft Excel software (Microsoft Software Inc., Redmond, WA, USA).

## 4. Conclusions

The findings of the present study demonstrated that pretreatment of HUVECs with GTE, lut, and lut-7-g inhibited the adhesion of THP-1 to TNF-α-activated HUVECs and cell surface expression of adhesion molecules in HUVECs in a dose-dependent manner. However, OA did not exhibit these effects. Decreased mRNA expression of ICAM-1 and VCAM-1 in TNF-α-activated HUVECs by GTE, lut, and lut-7-g reveal their efficacy in suppressing the expression of adhesion molecules at the transcriptional level. Furthermore, GTE, lut, and lut-7-g were shown to block the TNF-α-induced degradation of IκB, an indicator of activation of NF-κB. This implied that GTE, lut, and lut-7-g suppressed the mRNA expression of adhesion molecules via blocking the activation and nuclear translocation of NF-κB. The present results illustrate the therapeutic potential of GTE in preventing atherosclerosis.

## Supplementary Materials

Supplementary materials can be accessed at: http://www.mdpi.com/1420-3049/20/09/16908/s1.
