# Supplementary material for: Glossogyne tenuifolia Extract Inhibits TNF-α-Induced Expression of Adhesion Molecules in Human Umbilical Vein Endothelial Cells via Blocking the NF-κB Signaling Pathway
Source: Molecules. 2015 Sep 17;20(9):16908–23. doi: 10.3390/molecules200916908 (PMC6332270; doi:10.3390/molecules200916908)
Supplement: Supplementary file 1 [file molecules-20-16908-s001.pdf]

Supplementary Materials

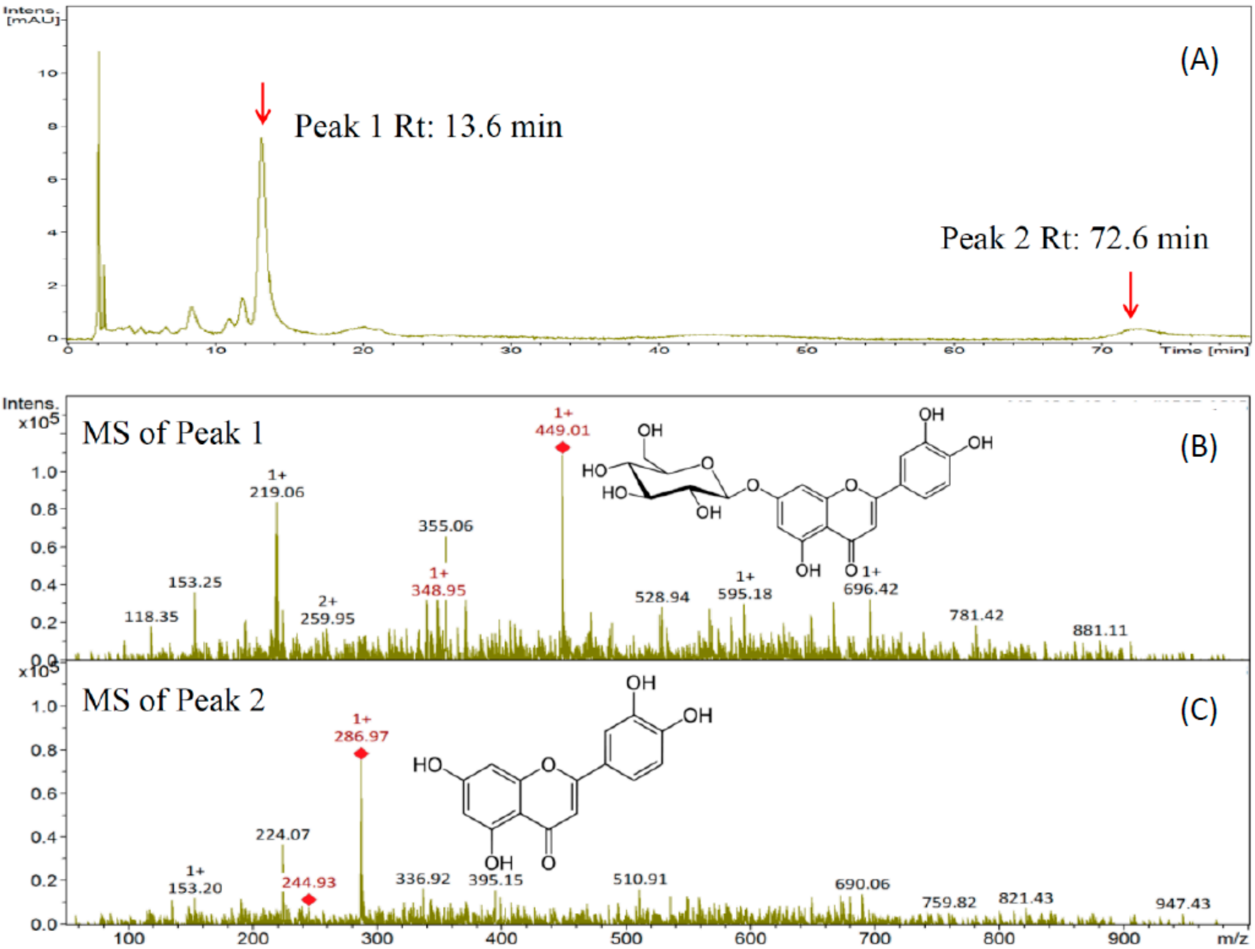

Figure S1. Chemical composition analyses of GTE. (**A**) HPLC chromatogram of GTE. The GTE sample was analyzed by an Ascentis™ C18 column (No. 581325-U, 5 µm, 250 × 4.6 mm; Supelco, Bellefonte, PA, USA) with HPLC (L-7100, Hitachi, Tokyo, Japan). A solution of methanol/0.05% acetic acid in water (40:60, *v*/*v*) was used as the mobile phase. The flow rate was 1.0 mL/min, and the detection was carried out at 350 nm; (**B**) UPLC-ESI-MS spectrometry profile of lut-7-g; and (**C**) UPLC-ESI-MS spectrometry profile of lut. UPLC-ESI-MS was performed with Doinex UltiMate 3000 UHPLC system (Thermo Scientific, Germering, Germany) tandem a Bruker amaZon SL system (Bruker Daltonik, Bremen, Germany). Separations were carried out using the same analytical column for HPLC analysis. The mass spectra were recorded using ESI in the positive mode, with ion spray voltage at 4500 eV, sheath gas pressure at 7.3 psi, capillary temperature at 180 °C and scanning from *m*/*z* 50 to 1000 amu. By selected ion detection at $[M + H]^+$ 287.0 and 449.0, the presences of lut and lut-7-g in the GTE were verified.

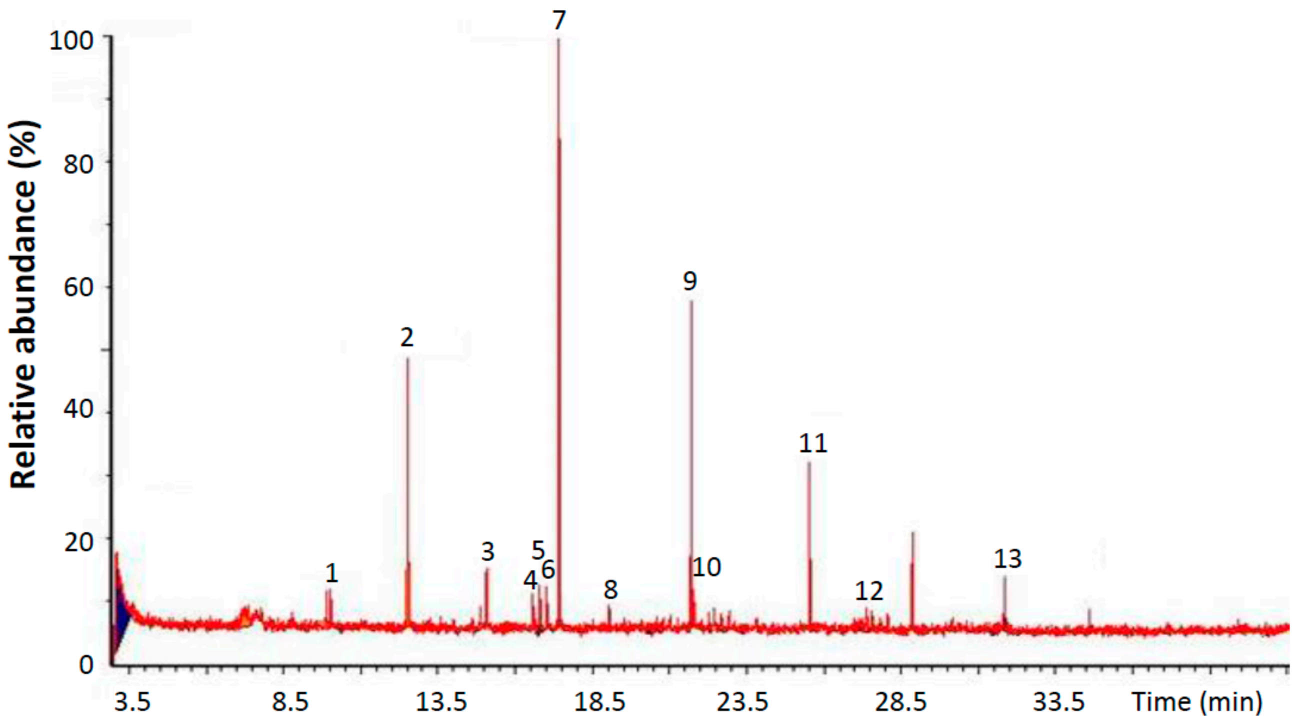

Figure S2. Gas chromatography-mass spectrometry profile of GTE. GC-MS analysis was performed using Clarus 500-GC and 500-MS system (Perkin Elmer, Waltham, MA, USA) with the electron impact mode (70 eV) injector, and a Clarus data system. The GC column was ZB-5 ms capillary column (30 m × 0.25 mm, film thickness 0.1 µm, Phenomenex, Torrance, CA, USA). Injector and detector temperatures were set at 300 °C and 280 °C, respectively. Oven temperature was kept at 140 °C for 5 min, then raised to 300 °C by a rate of 5 °C/min, kept at 300 °C for 10 min. The carrier gas was helium at a flow rate of 1 mL/min. Diluted samples of 1.0 µL were injected manually and in the splitless mode. The percentages of the compounds were calculated by the area normalization method. The components were identified by comparison of their mass spectra with the NIST MS 2.0 database (Gaithersburg, MD, USA).

Table S1. Chemical compositions of GTE analyzed by GC-MS.

No.	Component	Rt [a]	Percentage (%) [b]
1	1-Octanesulfonyl chloride	10.02	3.9
2	Trimethylsilyl 2,6-bis[(trimethylsilyl)oxy]benzoate	12.53	13.6
3	Agrinine	15.08	2.7
4	3,7-Dimethyloctyl trifluoroacetate	16.58	1.7
5	Tridecyl trichloroacetate	16.81	3.1
6	Dodecyl dichloroacetate	17.03	1.9
7	7-Chloro-5-phenyl-1-(trimethylsilyl)-1,3-dihydro-2*H*-1,4-benzodiazepin-2-one	17.41	31.0
8	Bis(2-ethylhexyl) (2*E*)-2-butenedioate	19.06	1.0
9	Trimethylsilyl 3-methyl-2-[(trimethylsilyl)oxy] benzoate	21.71	13.9
10	trans-2,3-Dimethoxycinnamic acid	21.79	1.3
11	Trimethylsilyl 2,4-bis[(trimethylsilyl)oxy]benzoate	25.53	8.1
12	1-Chloro-7-heptadecene	27.39	1.7
13	Trimethylsilyl 3-methyl-2-[(trimethylsilyl)oxy]benzoate	31.85	2.7

[a] Retention time (min); [b] Relative percentage calculated by integrated peak area.
